# Brain hubs in lesion models: Predicting functional network topology with lesion patterns in patients

**DOI:** 10.1038/s41598-017-17886-x

**Published:** 2017-12-20

**Authors:** Binke Yuan, Yuxing Fang, Zaizhu Han, Luping Song, Yong He, Yanchao Bi

**Affiliations:** 1 National Key Laboratory of Cognitive Neuroscience and Learning and IDG/McGovern Institute for Brain Research, Beijing Normal University, Beijing, 100875 China; 2 Department of Neurology, China Rehabilitation Research Center, Rehabilitation College of Capital Medical University, Beijing, 100068 China

## Abstract

Various important topological properties of healthy brain connectome have recently been identified. However, the manner in which brain lesion changes the functional network topology is unknown. We examined how critical specific brain areas are in the maintenance of network topology using multivariate support vector regression analysis on brain structural and resting-state functional imaging data in 96 patients with brain damages. Patients’ cortical lesion distribution patterns could significantly predict the functional network topology and a set of regions with significant weights in the prediction models were identified as “lesion hubs”. Intriguingly, we found two different types of lesion hubs, whose lesions associated with changes of network topology towards relatively different directions, being either more integrated (global) or more segregated (local), and correspond to hubs identified in healthy functional network in complex manners. Our results pose further important questions about the potential dynamics of the functional brain network after brain damage.

## Introduction

The human brain comprises highly interconnected units on multiple scales. At the macroscale level, the functional connectivity between regions can be measured as the temporal correlation of time courses of blood oxygen level-dependent fluctuations of functional magnetic resonance imaging^1^. The human whole-brain functional network can be constructed based on inter-regional functional connectivity and its topological properties can be studied with graph theory approaches^2,3^. One of the major findings in such “connectomic” literature in the past decade is the identification of a set of brain regions that are thought to play more important roles in the network communication than others, which tend to be implicated in various types of degenerative disease^4,5^. These regions are often considered the brain “hubs”^6^.

The “importance” of a brain region in the functional network structure has been considered from different aspects. One of the most common ways is to simply look at the number and/or strength of functional connections a region has (i.e., degree centrality). This approach has consistently identified the posterior cingulate cortex/precuneus (PCC/PCu), the medial prefrontal cortex (mPFC) and the inferior parietal gyrus as “hubs”^7–9^. Other methods have been proposed to define hub regions that take into consideration other topological aspects of the network such as modular structure^10–13^, considering regions that are the most important in linking different functional modules as connector hubs. These connector hubs, defined on the basis of “participation coefficient” (PC)^14^, i.e., the proportion of the number of a region’s inter-module connections to its total connections, mainly distributed in the anterior insula, the bilateral middle frontal gyrus (MFG), the bilateral precentral gyrus, the dorsal mPFC, and the superior parietal cortex.

A critical alternative approach to determining the importance of a region in network communication is to examine how much the network properties are altered if a given region is damaged. In patients with brain lesions, Gratton *et al*.^11^ found that the mean lesion severity (the nodal lesion percentages scaled by the region’s centrality measure in the healthy group) of regions with high PCs, not those with high within-module degree (WMD)^14^, was significantly correlated with the modularity property of the patient’s functional network. Such results offer compelling evidence that regions with higher PCs are more indispensable in maintaining a functional network’s modularity and, hence, are more important hubs. Nevertheless, many important issues remain to be addressed. First, brain lesion is neither comparable nor independent across regions^15,16^. The distribution of brain lesions is complexly constrained by vascular properties and should be understood as a high-dimensional multivariate pattern, which cannot be adequately addressed by the univariate or multiple linear regression approaches. Second, this study did not test the impact of lesion for specific regions. Furthermore, the changing directions of network topology after lesions have been shown to be complex^17^, and lesions of different regions might lead to opposite direction of changes on the small-worldness – the balance between the integration and segregation – of the remaining network^18,19^. For instance, Sporns *et al*.^18^ showed that simulated lesions in connector hubs increase the distance among modules and led to increased clustering coefficient and small-worldness, while simulated lesions in within-module hubs decrease these measures. Given that simulation lesion studies^10,20,21^ relies on specific assumptions about the mechanisms of attack, which does not necessarily reflect actual brain lesion patterns, studies based on real brain damage data that employ multi-variate approaches are needed to understand how lesion encompassing various brain regions affect functional network topology.

**Figure 1:**
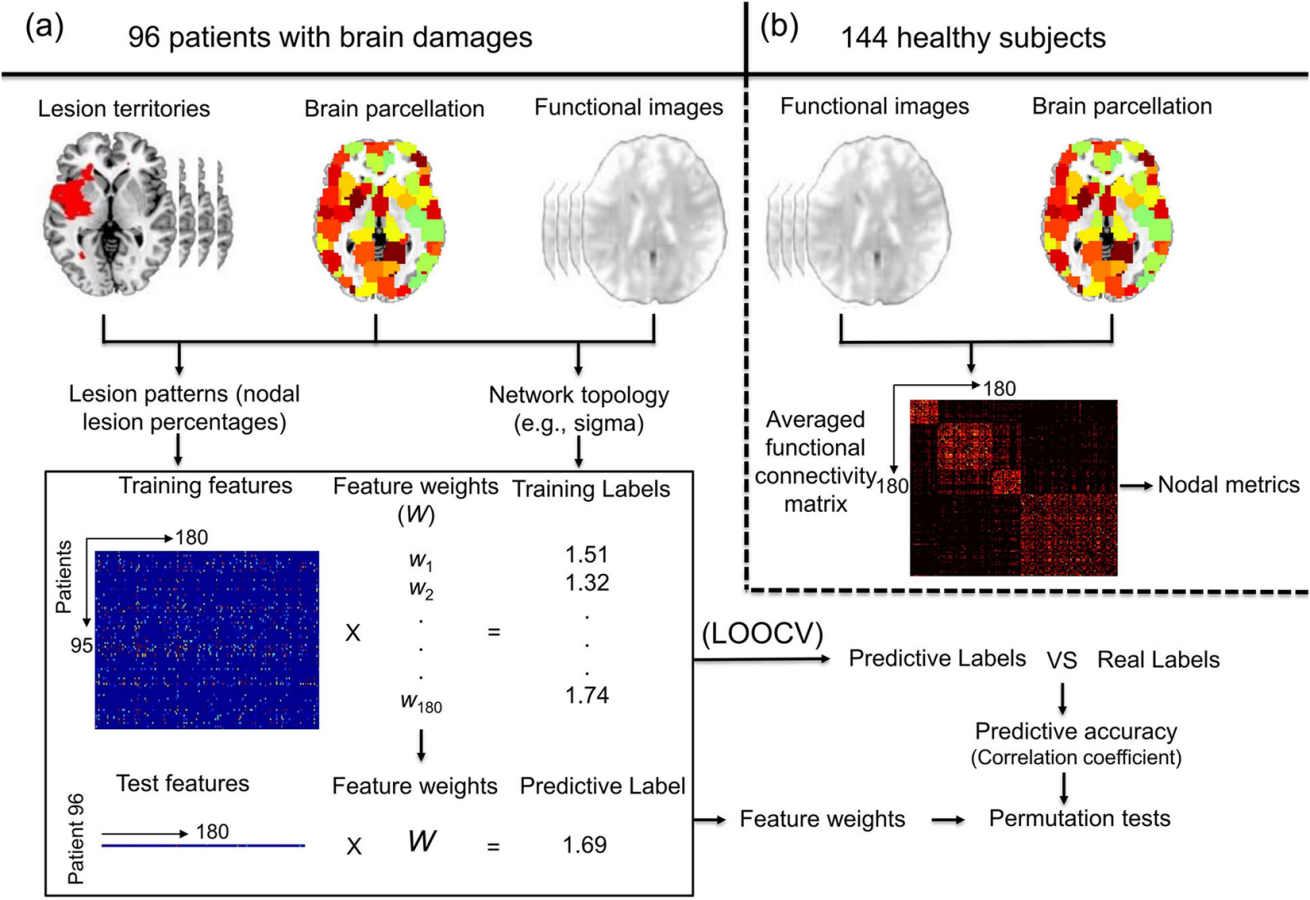
Overview of the methods to detect lesion hubs. (**a**) First, lesion pattern (i.e., lesion percentages of nodes) and network topology of the binary functional network were calculated for each of the 96 patients. Then, a lesion model using SVR was trained with lesion patterns of 95 patients as features, and network topological measures as training labels. Next, the trained model were used to predict the network topology of the test patient with his or her lesion pattern. A nested LOOCV was applied and the prediction accuracy of each lesion model was evaluated by calculating the Pearson correlation coefficient between the predicted labels and real labels. After 1000 permutation tests, the model significance was calculated. For the lesion model which could be significantly predicted by lesion patterns, features with significant weights were extracted, and considered as “lesion hubs”. (**b**) The averaged functional network of the 144 healthy participants was used to construct a healthy connectome, on which modularity analyses were performed and nodal metrics were calculated.

We applied a multivariate support vector regression (SVR) approach to evaluate the relationship between brain lesion patterns (i.e., the pattern of lesion extent across various cerebrum nodes) and whole-brain functional network topological properties in 96 patients with brain damage (Fig. 1). In contrast to univariate approaches, where each region is examined separately (e.g., linear regression), SVR involves a linear regression analysis in a high-dimensional feature space (here, lesion percentage in each region as features) to make continuous measurement predictions^22–24^. The SVR model yields weights for each region, indexing the extent to which the region’s lesion contributes to the prediction of different topological properties^15,16^. Regions with higher predictive weights indicate that their damage would lead to greater changes of the functional network and are more indispensable. These regions are thus considered “lesion hubs”. Finally, we compared these lesion hubs to those hubs conventionally defined by graph attributes in the functional network in a healthy population to understand the relationship between these two perspectives of delineating brain network properties.

## Results

Ninety-six patients (19 females, mean age ± SD = 44.56 years ± 13.21, range 19–74 years) with focal brain damages in sub-acute and chronic phase were analyzed (see Table S1 for details). The Craddock parcellation^25^ containing 180 cerebrum nodes were adopted and for each patient the lesion percentage in each node was calculated (see Figure S1 for lesion overlap map). All but two nodes have at least one patient with damaged voxels. The lesion percentages of the 178 nodes were used as features for the linear SVR^24^ analysis to predict functional network topological properties.

### Lesion pattern significantly predicted damaged network topological properties

For functional network topological properties, we first considered small-worldness (sigma), a composite measure about the extent of optimal balance of functional integration and segregation, then in the subsequent analyses looked at network global efficiency (network gE) and network local efficiency (network locE) separately to understand the direction of lesion-induced changes in the context of affecting the small-worldness. We calculated these topological properties in a single threshold (sparsity, *s* = 0.15) in the main analyses and validated the results by calculating the cumulative topological properties in the full range of network sparsity using the area under curve (AUC) method^26^. Leave-one-out-cross-validation (LOOCV) were used to calculate the prediction accuracy (the Pearson correlation coefficient between the predicted and actual labels) and significance level was computed based on 1000 permutation tests^23^. Note that we also examined models where the total lesion volume was included as an additional predictor. The rationale was lesion pattern might be affected by total lesion volume, and if it was the lesion volume that was the actual significant predictor for network topology, rather than the lesion pattern (specific lesion distribution), in the SVR model its contribution should be significant and the lesion regional features would not.

Permutation test found that the whole-brain lesion pattern, i.e., the distribution pattern of the lesion across the 178 nodes, predicted functional network small-world sigma with significantly above-chance accuracies (*r* = 0.223, *P*one-tailed = 0.025). In flow-up analyses to test the direction (integration or segregation) that contributes to the sigma changes, we found that the whole-brain lesion pattern significantly predicted network gE (*r* = 0.269, *P*one-tailed = 0.015) and network locE (*r* = 0.220, *P*one-tailed = 0.041). Figure S2 showed the scatter plots of the actual network topological property values and the predicted values using these three significant lesion models and the corresponding linear regression lines. Without the total lesion volume as a feature, significant above-chance accuracies were observed for small-world sigma (*r* = 0.225, *P*one-tailed = 0.024) and network gE (*r* = 0.268, *P*one-tailed = 0.019), with a positive trend for network locE (*r* = 0.183, *P*one-tailed = 0.055). That is, the performances of these lesion models were minimally affected after adding the total lesion volume as one additional feature.

A split-half analysis was also conducted to validate the effectiveness of the lesion models. We randomly divided the patients into two subgroups and then performed SVR prediction in each subgroup. The results were relatively reliable: For small-world sigma, the significant prediction was observed in subgroup2 (subgroup1: *r* = 0.185, *P*one-tailed = 0.091; subgroup2: *r* = 0.425, *P*one-tailed = 0.013). Significant predictions were observed in both subgroups for network gE (subgroup1: *r* = 0.383, *P*one-tailed = 0.006; subgroup2: *r* = 0.402, *P*one-tailed = 0.007), and network locE (subgroup1: *r* = 0.348, *P*one-tailed = 0.018; subgroup2: *r* = 0.392, *P*one-tailed = 0.013).

### Lesion hubs: Regions showing significantly predictive effects to network topology

**Figure 2:**
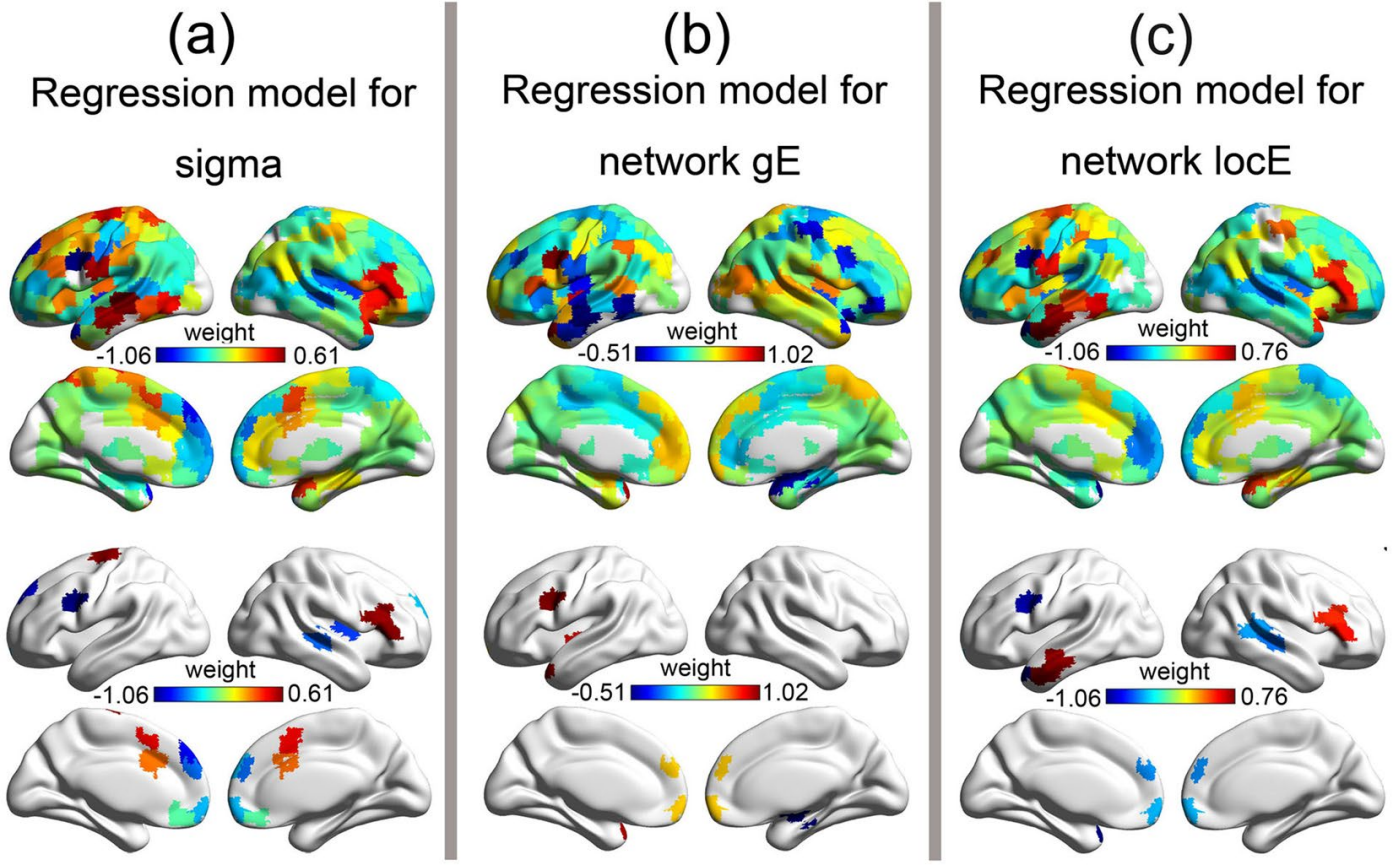
Feature weights and lesion hubs in significant lesion models. The first row shows the distributions of feature weights in the three significant lesion models. The second row shows the lesion hubs with significant feature weights (*P* < 0.05, two-tailed, permutation test) in each of the three significant lesion models. Cold colors represent negative weights and warm colors indicate positive weights.

The predictive weight of each feature was obtained by training a SVR model using all patients. The unthresholded maps of the feature weights in the three regression models are shown in Fig. 2 (first row). Features with significantly above-chance (*P* < 0.05, permutation test, two-tailed) weights were extracted (Fig. 2, second row). The weight of the total lesion volume did not reach significant in any models. As shown in Fig. 2 and Table 1, the following regions showed significantly negative weights in the prediction models for network locE and sigma and significantly positive weights in the prediction model for network gE: the left MFG, the bilateral superior frontal gyrus, and the orbital frontal pole. Clusters in the right superior temporal gyrus (STG; extending into the supramarginal gyrus), the superior portion of the left temporal pole (sTP), the bilateral superior frontal gyrus (SFG), the bilateral mPFC and the bilateral insula also showed this pattern, although their effects did not reach significance in all three models.

**Table 1:** Nodal attributes in the healthy functional network of lesion hubs.

Name of ‘winning region’	MNI (center voxel)	WMD	PC	gE (Z-score)	Module	Lesion model
**Integration lesion effect to network topology**						
L MFG (34)	−46, 10, 31	−0.34	**0.56**	**0.69**	FPN	N_locE (−), N_gE (+), sigma (−)
SFG (91)	0, 54, 25	**1.02**	0.06	**0.49**	DMN	N_locE (−), N_gE (+), sigma (−)
FP orb (109)	1, 58, −8	**1.12**	0.06	**0.43**	DMN	N_locE (−), N_gE (+), sigma (−)
L sTP (78)	−42, 15, −29	**1.22**	0.10	**0.7**	DMN	N_locE (−), N_gE (+)
R STG (107)	54, −27, −1	−0.27	**0.43**	**0.28**	DMN	N_locE (−), sigma (−)
L insula (4)	−38, −12, −3	**0.77**	0.10	**1.2**	SSN	N_gE (+)
R STG (69)	59, −42, 10	−0.69	**0.36**	**0.39**	SSN	N_locE (−)
L SFG (133)	−10, −49, 39	**0.82**	0.20	**0.45**	DMN	sigma (−)
R SFG (193)	14, 58, 28	**1.52**	0.05	**0.79**	DMN	sigma (−)
mPFC (51)	−1, 41, −13	**1.62**	0.05	**0.98**	DMN	sigma (−)
R insula (35)	40, −3, 10	**1.13**	0	**0.89**	SSN	sigma (−)
**Segregation lesion effect to network topology**						
R IFG tri (144)	51, 30, 4	−0.42	**0.43**	**0.68**	SSN	N_locE (+), sigma (+)
R IFG oper (164)	51, 23, 20	−0.87	**0.49**	**0.39**	SSN	N_locE (+), sigma (+)
R hippocampus (155)	20, −10, −17	−0.37	0.09	-0.7	DMN	N_gE (−)
L aMTG (72)	−58, −13, −17	**1.12**	0.15	**0.59**	DMN	N_locE (+)
L aMTG (101)	−52, −2, −29	**0.72**	0.12	**0.20**	DMN	N_locE (+)
paraCC (13)	2, 14, 48	**0.67**	0.20	**1.03**	SSN	sigma (+)
dACC (55)	0, 18, 32	**0.58**	0.20	**1.21**	SSN	sigma (+)
L precentral (90)	−18, −14, 69	**0.58**	0	**0.16**	SSN	sigma (+)

L: left hemisphere; R: right hemisphere; MFG: middle frontal gyrus; SFG: superior frontal gyrus; FP orb: orbital frontal pole; sTP: superior portion of the temporal pole; STG: superior temporal gyrus; mPFC: medial prefrontal cortex; IFG tri: pars triangularis of the inferior frontal gyrus; IFG oper: pars opercularis of the inferior frontal gyrus; aMTG: anterior middle temporal gyrus; paraCC: paracingulate cortex; dACC: dorsal anterior cingulate gyrus. The anatomical regions of a node was labeled according to its overlap with the Harvard-Oxford Atlas^67^ (112 regions with a 25% probability threshold). The number in the brackets is the number of the brain region in the brain parcellation (Craddock parcellation, *N* = 200). Bold font indicates that the lesion hub also has a relatively high nodal metric in the healthy connectome (PC > 0.3, WMD > 0 and gE_Z-score_ > 0). DMN: default mode network; FPN: frontal-parietal network; SSN: somatosensory network. N_gE: network gE; N_locE: network locE. +: positive weight; -: negative weight.

There were also regions showing the opposite pattern, with positive weights for the lesion models of network locE and sigma and/or negative weights for network gE: The pars opercularis and pars triangularis of the right inferior frontal gyrus (IFG oper and IFG tri) had significantly positive weights for network locE and sigma; The left anterior middle temporal gyrus (aMTG, extending to the middle part of MTG) had significantly positive weights for network locE; The right hippocampus had significantly negative weight for network gE; The bilateral paracingulate cortex (paraCC), the bilateral dorsal anterior cingulate gyrus (dACC) and the left precentral gyrus had significantly positive weights for sigma. The feature of the total lesion volume did not show significant weight in any lesion model.

**Figure 3:**
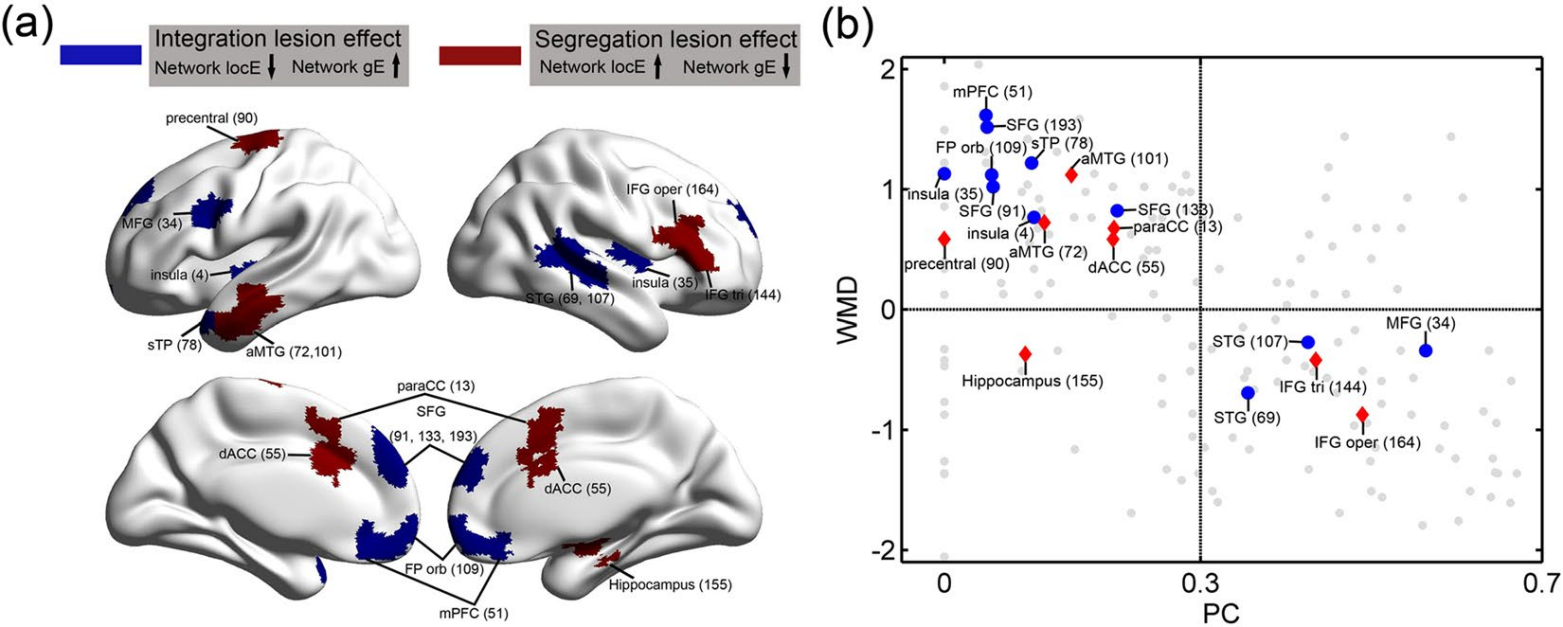
Correspondence between the two classes of lesion hubs and hub regions in a healthy connectome. (**a**) Brain regions whose lesions show significant integration lesion effect (blue) and segregation lesion effect (red) to network topology in the three significant lesion models. The number in the brackets is the number of the node in the brain parcellation^25^. (**b**) The PC and WMD values in the healthy connectome. Blue circle indicates a region showing integration lesion effect, red rectangle indicates a region showing segregation lesion effect, and grey dot indicates the nodal roles of the rest brain regions. For the abbreviations of the regions, see Table 1.

To incorporate lesion hubs obtained from the three graph measures, we plotted all significant regions from these models in Fig. 3a, with two colors corresponding to the two classes, i.e., those whose lesion induced more integrated/global processing (reduced network locE or sigma and/or increased network gE), and those whose lesion induced more segregated/local processing (increased network locE or sigma and/or reduced network locE).

### Relationship between lesion-model feature weights and healthy connectome nodal properties

We constructed a healthy functional network using the same Craddock atlas (*n* = 180) by averaging the Fisher z-transformed functional connectivity matrices of 144 healthy adults in a published database^27^ (see detailed information of the healthy adults, the conventional preprocessing procedures in the supplementary materials). Modularity analysis^28^ based on a spectral optimization algorithm was performed to reveal the modular structure of the healthy network. To validate the robustness of the modular assignment, multiple sparsity thresholds (0.05–0.2, step 0.01) were analyzed. Four nonoverlaping modules, including the default mode network (DMN), the frontal-parietal network (FPN) and the somatosensory network (SSN) and visual network were consistently identified (Fig. S3a and b). We then chose a representative sparsity, *s* = 0.15 to check the modular assignment (Fig. S3c and d) and calculated the nodal properties. The lesion hubs were distributed in DMN, FPN and SSN in the healthy network (Table 1).

The hub distributions in the healthy network were analyzed based on three nodal metrics: participant coefficient (PC) and within-module degree (WMD), reflecting nodal roles in connecting different modules and within each module, respectively, and nodal gE, measuring global information communication efficiency. The distributions for each of the three nodal metrics are shown in Figs S3(e,f,g) and S4, and the regions showing relatively high nodal properties (PC > 0.3, WMD > 0, nodal gE_*Z*-score_ > 0) are in accordance with findings in the literature.

The two classes of lesion hubs tended to exhibit high nodal metrics in the healthy connectome. As shown in Table 1 and Fig. 3b, lesion hubs that are of the “integration effect” class in the left MFG and the right STG, and lesion hubs of the “segregation effect” class in the right IFG tri and IFG oper, were connectors with high nodal PC (> 0.3). The other lesion hubs belonging to either class were provincial hubs with high nodal WMD (> 0): The “integration effect” lesion hubs in the bilateral SFG, the bilateral FP orb, the left sTP, the bilateral mPFC and the bilateral insula, and the “segregation effect” lesion hubs in the left aMTG, the bilateral dACC, the bilateral paraCC and the left precentral gyrus. All lesion hubs (except the right hippocampus) also showed high nodal gE (*Z*-score > 0).

Intriguingly, there are also regions with high nodal metrics in the healthy connectome that did not show significantly weights in the lesion models: the PCC/PCu, the lateral parietal cortex, the dorsolateral prefrontal cortex (DLPFC).

### Results of the validation analyses

A series of validation analyses were performed to examine the robustness of the main results by using different network construction procedures (sparsity thresholds; head motion treatment; global signal removal; parcellation scheme) or participant selection methods (right-handed stroke patients, only male patients). The prediction accuracies of the validation analyses are summarized in Table S3. Details are described below.

#### The effects of the network sparsity thresholds

To consider the results across a broad sparsity range, we calculated the cumulative values of the network metrics by using the AUC^26^ for each network metric across the sparsities of 0.13–0.47 (step 0.01, see Method for the identification method of the sparsity range), to avoid potential bias in selecting a specific sparsity. Using such AUC metrics as labels, the predictive accuracies for the three lesion models remained significant (sigma: *r* = 0.29, *Pone-tailed* = 0.013, network locE: *r* = 0.317, *Pone-tailed* = 0.007, and network gE: *r* = 0.287, *Pone-tailed* = 0.012). The brain regions with significant weights in these three significant lesion models were nearly identical to those in the main results (Fig. S5).

#### Motion scrubbing

After the “scrubbing” procedure was performed on the preprocessed images to further deal with head motion, one patient was excluded because the number of remaining volumes was fewer than 50. Significant prediction were observed for sigma (*r* = 0.241, *Pone-tailed* = 0.033), network locE (*r* = 0.244, *Pone-tailed* = 0.019) and network gE (*r* = 0.246, *Pone-tailed* = 0.024) were still significant. In the three significant lesion models, the distributions of the brain regions with significant feature weights were largely consistent with the main results (Fig. S6).

#### The effect of global signal regression (GSR)

We perform GSR to reduce the motion-induced noises^29^ in the main analysis. When the global signals were not removed, the predictive accuracy was significant in the lesion model for network locE (*r* = 0.249, *Pone-tailed* = 0.019), and not for network gE (*r* = 0.168, *Pone-tailed* = 0.067) or sigma (*r* = 0.093, *Pone-tailed* = 0.136). For network locE, the same lesion hubs to those in the main results were obtained: the left sTP and the right STG with negative weights and the left aMTG with positive weight.

#### The effect of brain parcellation

We repeated the whole analyses using another brain parcellation – Brainnetome Atlas^30^ (246 nodes) – to investigate whether the results are affected by the choice of parcellation scheme. We chose this atlas because its parcellation was done on the basis of structural connectivity and had comparable number of regions with the Craddock 200 atlas. We also considered a third atlas, Craddock atlas with 1000 parcellations^25^, which contained much finer parcellations and could help to refine the main findings. When using the Brainnetome atlas, all three lesion models were still significant, with better performance than the main results: for sigma, *r* = 0.449, *Pone-tailed* < 0.001; for network gE, *r* = 0.454, *Pone-tailed* < 0.001; for network locE, *r* = 0.387, *Pone-tailed* = 0.002. Features with significant weights that covered the same brain regions (e.g., the left MFG, the left TP, the right IFG and the left precentral gyrus) and showing the same type of lesion effects as those in the main results were observed (Fig. S7). While other lesion hubs detected using Craddock 200, including the left mPFC, the left FP and the right insula and the bilateral dACC, did not reach significance in the model using the Brainnetome Atlas, they nonetheless had relative greater weights than the average (top 50%). When using the “Craddock 1000 atlas”^25^, lesion pattern still predicted functional network small-world sigma (in sparsity threshold, *s* = 0.01, an arbitrary threshold to ensure sparse and fully-connected networks, Table S3) with above-chance accuracies (*r* = 0.188, 1000 permutation tests *P*one-tailed = 0.045), as well as network gE (*r* = 0.267, *P*one-tailed = 0.016) and network locE (*r* = 0.319, *P*one-tailed = 0.004). The significant contributing features (brain regions, Fig. S8) aligned more differently from the other two atlases (Craddock 200 and Brainnetome). The differences for the significant predictor brain regions across atlases might be because using a finer brain parcellation with too many features (1000 partitions), correlations among features are much higher (the percentage of nonindependent features (*r* > 0.3) was 0.18 for Craddock 200 and 0.33 for Craddock 1000). Although SVR does not have assumptions regarding independence between features, feature weights on correlated features would be shrunk proportionally to the number of correlated features^31,32^. Thus lesion hubs that are damaged together with others may be compromised in the SVR model. We thus focused on the lesion-hub detection results with the two atlases with comparable number of regions.

#### The effects of lesion type and handedness

To test the extent to which our results are affected by etiology and handedness, we re-performed the SVR prediction and lesion hub detection using only the 76 right-handed (handedness assessed using the Chinese adaptation of handedness inventory^33^) stroke patients (13 females; age: mean ± SD = 47.01 ± 11.22; range, 20–74 years). The predictive accuracy was significant in the lesion model for network gE (*r* = 0.222, *Pone-tailed* = 0.034). There was a trend of effect for network locE (*r* = 0.199, *P* = 0.051) and not for sigma (*r* = 0.049, *Pone-tailed* = 0.194). Lesion hubs that predicted network gE were the same as those in the main results: the left IFG, the left insula and the bilateral SFG with positive weights, the left aMTG with negative weight.

#### The effect of gender

The ratio of male/female in the patient group is unbalanced (77/19). To test whether our results were affected by gender, we re-performed the SVR prediction and lesion hub detection using only the male patients (age: mean ± SD = 47.01 ± 11.22; range, 20–74 years). The predictive accuracy was significant in the lesion model for network gE (*r* = 0.246, *P*one-tailed = 0.03) and locE (*r* = 0.278, *P*one-tailed = 0.02). No effect was observed for sigma (*r* = 0.106, *P*one-tailed = 0.117). Lesion hubs that predicted network gE and locE were the same as those in the main results: The left MFG and the left insula.

#### Validating the feature signs using single-nodal lesion patients

Given that the interpretation of feature signs in multivariate analyses requires caution (see Discussion), we further carried out an analysis using patients whose lesion largely only affected one node (the left insula, *N* = 11, Fig. S9) to validate the implications of the feature signs (i.e., whether positive or negative features imply increased or reduced topological values). This was the lesion hub obtained in the main analyses that has the largest number of patients with relatively contained lesion allowing for this univariate analysis. We compared the network topological properties between these 11 patients and their age- and education-matched controls (*N* = 36, Table S4), and found that the network locE significantly decreased in patients with the left insula lesion (*P* < 0.05, false discovery rate correction, FDR) across a wide sparsity range (0.01–0.45, i.e., the minimum sparsity to the maximum sparsity that retained all positive edges for all subjects in both groups, step 0.01) and the network gE tended to increase in these patients (Fig. S10). These results were consistent with the “integration” effect of the left insula showed by the SVR models.

## Discussion

Using multivariate pattern regression analysis based on brain structural lesions and resting-state functional imaging in 96 patients with brain damage, we established that the lesion patterns of patients could robustly predict the whole-brain functional network topology. Two types of lesion hubs, i.e., those brain regions whose damage associated with two directions of topological change (more integrated/global vs. more segregated/local), were identified. All lesion hubs (except the right hippocampus) also showed high centrality in the healthy functional connectome, but showed complex patterns of correspondence with different type of healthy centrality measures, with both lesion-hub classes containing regions with high PC and also regions with high WMD. There were also hubs in the healthy connectome that were not observed as lesion hubs.

Different from previous studies about the effects of lesion in functional network topology using univariate methods^11,13,20,21^ (see a review by Aerts *et al*. ref.^17^), we used a more appropriate (multivariate) approach for real brain damage data that takes into consideration of the complex interaction of lesion patterns across brain regions. We established that aspects of the integration and segregation properties of functional network topological characteristics can be predicted from the pattern of brain lesions, including small-worldness (the balance between integration and segregation), network global (relative integration) and local efficiency (relative segregation). Such effects of lesion distribution patterns were not explained by the overall extent of lesion because the total lesion volume was not a significant predictor for topology in the SVR model. That is, it is not the sheer size of the lesion but the distribution pattern of the lesion that matters for the network’s integrity.

The multivariate lesion models also provide the specific manner in which their lesion changes the functional network topology (i.e., the feature weights and sign). Our results revealed two distinct classes of lesion-hubs, whose lesions cause the whole functional network to be either significantly more integrated (lower network locE or sigma and/or higher network gE) or significantly more segregated (higher network locE or sigma and/or lower network gE). All lesion-hub regions (except the right hippocampus) in these two classes show relatively high centrality measures in healthy functional connectome, indicating the overall correspondence between different approaches in identifying regional importance in the network topology. The manner of correspondence, however, is rather complex, with both classes of lesion hubs containing both regions with high PC (i.e., connectors between different systems) and regions with high WMD (i.e., provincial hubs within specific systems). Below, we discuss these two-class lesion hubs in the context of their status in the healthy functional connectome in greater details.

### Lesion-hubs with relative “integrated-topology” lesion effect

Some of this type of lesion hubs have high WMD in healthy connectome: the bilateral SFG, the bilateral FP orb, the left sTP, the bilateral mPFC and the bilateral insula. These regions have been consistently identified as hub regions in previous studies using the degree-based approaches, with all regions except the insula belonging to the so-called DMN system, which is more active during rest and is deactivated during explicit tasks^7,8,10,12,34,35^. That is, these regions have the greatest number and/or strength of functional connections with other regions in the healthy system. Physiological studies have shown that regions with a high degree, including this type of lesion hubs, tend to have high biological cost, with high rates of cerebral blood flow, aerobic glycolysis and oxidative glucose metabolism^9,36–38^. Also, their changes are strongly associated with various types of neurological disorders^39,40^. The insula was identified as a provincial hub with high WMD and gE in the current study, and previous studies have regarded it as a connector with high PC^12^ or a rich-club region^41^ and participating a wide range of cognitive functions^42,43^. Our findings provide direct evidence for the necessity role of these regions in the organization of the whole-brain functional network.

Other lesion-hubs that have the “integrated-topology effect”, the left MFG and the right STG, have high nodal PC in the healthy connectome, meaning that they have particularly more and/or stronger connections across different modules and are thus likely to integrate information from different functional systems^42,43^. The left MFG connects FPN and SSN, the right STG connects DMN and SSN (Fig. S2). The MFG node also contained large part (44%) of the left inferior frontal junction, a critical node for cognitive control^44^. Complementing previous studies using univariate lesion or simulated-lesion methods^10–12^, we showed that damaging these regions indeed strongly affects the functional network’s communication efficiency and the balance between integration and segregation, which may lead to widespread cognitive deficits^13^.

### Lesion hubs with relative “segregated-topology” lesion effect

Some of this type of lesion hubs having high WMD in the healthy connectome (the bilateral dACC, the bilateral paraCC, the left precentral gyrus and the left aMTG), while others have high nodal PC (the right IFG tri and IFG oper). The dACC, including the paraCC, monitors performance and signals the need for behavioral adaptation and is important to change behavior^45^. The left precentral gyrus is one of the primary motor regions and has been identified as a connector in a functional connectome^10^. The left aMTG belongs to the DMN and is a core region in the semantic system which bridges the memory based simulation system and the language-based semantic system^46^. The right IFG connects SSN, FPN and DMN, and has been shown to be a connector^12^ that participates in a wide range of cognitive functions^42,43^. The right hippocampus has been identified as a connector^10^ but does not show either high nodal PC or WMD in the current study.

Together we observed a complex correspondence between hubs in lesion models and those in the healthy system–The damages of the same type of hubs in the healthy connectome may cause the network topology to change towards different directions. This pattern is different from the simple correspondence (integrating effect of connector “lesion” and segregating lesion effect of provincial hub “lesion”) revealed in some simulating lesion study^18^, highlight the need to study the dynamic changes of functional networks upon brain damage. One potential mechanism is the connectional diaschisis^21,47,48^. Besides disconnections induced by the lesion, there may be a considerable number of increased functional connections that over-compensate for the brain damage to the functional connectome^49^. Future work is clearly desired to understand the different types of dynamics induced by lesion patterns encompassing different hub regions.

The dynamic changes of functional connection patterns among intact regions due to lesions elsewhere may also explain the existence of brain regions that showed high nodal WMD, gE or PC in the healthy connectome but did not have significant feature weights in the lesion models, including the posterior part of the DMN (e.g., PCC/PCu and lateral parietal cortex) and the right DLPFC. That is, although they are densely connected in the healthy network, their involvements in the brain lesion are not significantly predictive of the network topology. These results do not seem to be compromised by the fMRI data quality. All nodes except the left mPFC (a lesion hub) satisfy the criteria for signal quality^50,51^ (mean tSNR > 80, Fig. S11). Furthermore, these results are difficult to be explained by false negatives due to a lack of statistical power, because many of these regions have a decent proportion of cases of being damaged or preserved in our patient population (e.g., 19/96 patients were damaged in the right DLPFC; Fig. S1). We speculate that this phenomenon might be related to the following aspects. First, the brain is likely to be wired with sufficient redundancy to increase resilience. Indeed, even after severe brain injury, the network topology (e.g., small-worldness) is well preserved, although less optimal^52,53^. In simulation studies, attacking a small portion of critical regions induced limited impacts on network topology, with the network integrity sharply dropping only after attacking 40% of degree-based hubs or 17% of connector hubs^10,54^. It is possible that the wiring redundancy is particularly pertinent to these regions, such that although they are normally well connected, routing possibilities for communication among other nodes are richer when they are no longer available because other regions are already connected. Another possibility is related to plasticity: when these regions are damaged, the rewiring among other regions is particularly efficient to compensate for the overall topological structure.

There are several methodological issues to note. A common methodological issue in patient studies is that the lesion pattern is constrained by vascular properties, with some regions tending to be more prone to brain damage than others (Fig. S1), which results in power differences among regions in some statistical testing. In contrast to univariate analysis, the method we employed, SVR, has no explicit assumptions regarding the nature of the data, such as the number of non-zero values in a feature vector, which is often required by univariate correlation or t-test analyses. It is nonetheless worth noting that in many cases, a region’s feature weight does not comply with the number of lesions: the FP orb (damaged in 4/96 patients), the left SFG (damaged in 3/96 patients), and the mPFC (damaged in 4/96 patients) were rarely damaged and yet were discovered to be lesion hubs. While multivariate approaches offer many advantages, the interpretation of individual feature requires caution. The feature weights, on which we relied to define hubs, were calculated in the context of the whole lesion patterns across patients. That is, it might be the complex patterns across the significant contributors that lead to the significant network changes. We thus chose to focus on those features that had significant weights as a group, considering them to have significant contributions to the functional network properties. The lack of simple characteristics of the hubs that were related to their segregating/integrating (local/global) role might be related to the multivariate information. Regarding the signs of feature weights, our univariate analyses using samples with single nodal lesion (the left insula) revealed topological changes in the same direction to that revealed by its sign in the SVR prediction model (integration effect). Henson *et al*.^19^ found that patients with focal hippocampus lesion also showed the same direction of results as our MVPA findings (i.e., integration lesion effect). These two data points suggest the functional relevance of positive and negative weights in our current lesion models, although certainly convergent evidence is desired. A further important question is whether the mechanisms in which brain damage affects functional network topology differs across etiology. It has recently been shown that a wide range of brain disorders tend to implicate hub regions^17,55^, the studies are far from conclusive about the finer patterns, especially complex direction of changes, as illustrated here. Finally, we have primarily focused on the topological properties relating to the small-worldness structure, and the integration and segregation hubs were defined in the context of the overall small-worldness measures. The lesion hubs that are more critical for the topological properties, such as the network modularity or the rich-club structure, remain to be carefully studied.

To conclude, by combining the lesion and functional imaging data of patients, we provided a new framework for detecting hubs using multivariate pattern regression analysis, where each patient’s overall lesion distribution was considered as a whole and nodal hubness was determined by the predictive contribution (weight) of its lesion to the whole-brain network topology. Our results revealed two different types of lesion-hubs, whose damage cause different manners of functional network topological changes, to either more segregated/local or more integrated/local topology, and both classes contain both connector hubs and provincial hubs in the healthy functional connectome. Our results highlight the necessary roles of specific regions in frontal and temporal cortices and the insula, in the maintenance of the optimized functional network topology, as well as the potential dynamic changes of the functional network after brain damage.

## Methods

### Participants

The inclusion criteria for patients were as follows: presenting with brain injury for the first time, at least one month post-onset, and no other neurological or psychiatric disease such as schizophrenia or alcohol abuse. Patients with a variety of etiologies (76 stroke patients, 16 trauma patients, 4 others; see Table S1 for details) were included in the main analyses to maximize lesion coverage. We also adopted a healthy dataset including 144 healthy participants to construct a mean healthy functional network. Note that this dataset was not chosen as a age/education-matched control group (See demographic information in the supplementary materials) to the patient group, but rather served to as a benchmark model to understand the correspondence between the hub distribution from the current patients’ data and that in a normative healthy system, which has been shown to be consistent across age and gender^12,56,57^.

All participants provided informed written consent. This study was approved by the Institutional Review Board of the National Key Laboratory of Cognitive Neuroscience and Learning, Beijing Normal University. All experiments were performed in accordance with relevant guidelines and regulations.

### Image acquisition of patients

Neuroimaging data for patients were collected on a 1.5 T GE SIGNA excite scanner (Milwaukee, US.).The participants lay supine with the head snugly fixed with straps and foam pads to minimize head movement. High-resolution T1-weighted 3D MPRAGE images in the sagittal plane were obtained with the following parameters: 248 slices, time repetition (TR) = 1226 ms, echo time (TE) = 4.2 ms, inversion time (TI) = 400 ms, flip angle (FA) = 15°, field of view (FOV) = 250 mm × 250 mm, matrix size = 512 × 512, slice thickness = 1.4 mm, gap = 0.7 mm, and voxel size after interpolation by scanner = 0.70 × 0.49 × 0.49 mm^3^. Two identical sequences of 3D T1 images were collected and averaged to improve the signal-to-noise ratio during analysis. Resting-state functional images along the AC–PC line were collected using the T2*-weighted echo planar image sequence with the following parameters: 28 axial slices, TR = 2000 ms, TE = 40 ms, FA = 90°, FOV = 210 mm × 210 mm, slice thickness = 4 mm, gap = 1 mm, duration = 4 min, and 120 volumes. During the resting-state scanning, participants were instructed to close their eyes, remain still, stay awake and not think about anything in particular. The FLAIR T2-weighted images, which had the same slice locations as the functional images on the axial plane, were collected with the following parameters: 28 slices, TR = 8002 ms, TE = 127.57 ms, TI = 2000 ms, FA = 90°, FOV = 250 mm × 250 mm, matrix size = 512 × 512, and voxel size = 0.49 × 0.49 × 5 mm^3^.

### Lesion mapping

The lesion mapping procedures have been described in detail in our previous studies^58,59^. Briefly here, the T2-weighted FLAIR images were co-registered to the averaged 3D T1 images. Visually referring to the T2-weighted FLAIR images, for each patient, the lesion mask was manually drawn on the averaged 3D T1 images slice by slice. Each patient’s averaged 3D T1 images were manually registered into the Talairach space. The affine transformation matrix between the native and Talairach spaces was extracted and then used to transform the lesion mask into the Talairach space. The lesion mask were finally transformed into the Montreal Neurological Institute (MNI) space by applying the affine transformation matrix from the Talairach template to the MNI template. The voxel-wise and node-wise lesion overlap map of the 96 patients was showed in Fig. S1.

### Construction and characterization of the patients’ functional network using graph theory

Only fMRI signals in intact voxels were considered in the following analyses.

#### Functional data preprocessing

Patient’s functional data underwent the following preprocessing steps: deletion of the first 10 volumes, slice timing and motion correction. No participant exhibited a head motion > 2 mm maximum translation or 2° rotation. The motion-corrected functional images were first co-registered to the averaged 3D T1 images via their FLAIR T2-weighted images and were then spatially normalized into the MNI space by applying the aforementioned two affine transformation matrices. The linear trend was removed, and the nuisance signals (Friston 24 head motion^60^, global signal, cerebrospinal fluid, and white matter) were linearly regressed out from each voxel’s time course. No spatial smoothing was conducted. Then, temporal band-pass filtering (0.01–0.1 Hz) was performed on the residuals.

#### Constructing the functional network

We adopted the Craddock parcellation^25^ for network construction because this parcellation scheme was developed based on resting-state functional connectivity homogeneity and our question was about functional network properties. We excluded the cerebellum and brainstem (20 nodes). Individual functional networks were constructed by calculating the Pearson’s correlation coefficients between pairwise nodes. The *r* values in each matrix were transformed to *z* values using Fisher’s r-to-z conversation and only positive correlation coefficients were retained following the convention.

Given that the network sparsity may affect the topological properties of the resulting network^26^, we validated the main results (using sparsity 0.15) by considering the network properties across a broad sparsity range using the AUC^26^ approach, which is a widely used measurement for multiple sparsity analyses in brain network studies^26,61^. That is, across a wide sparsity range, network metrics were calculated with a step of 0.01, forming a curve of network metric values. The area under that curve was calculated as the integrated measure of this network metric, using the formula ${E}_{{\rm{i}}{\rm{n}}{\rm{t}}{\rm{e}}{\rm{g}}{\rm{r}}{\rm{a}}{\rm{t}}{\rm{e}}{\rm{d}}}={\int }_{s1}^{s2}E{d}_{s}$, where the *s*1 and *s*2 are the lower and upper sparsity thresholds. The minimum network sparsity ensured that each patient’s network was fully connected (*s* = 0.13); the maximum network sparsity was that retained all positive correlative coefficients in the network (≥ 0.47 across the 96 patients).

Brain lesion generally disrupted both of the functional connectivity strength and network topology. Given our purpose to assess the nodal hubness based on its lesion impact (weight) to pure network topology, we used the binary thresholded networks (1 for presence of connection and 0 for absence) for further analyses.

#### Characterizing the functional network using graph theory

For small-worldness, we used a more biologically sensible metric than the conventional approaches^62^– the small-world index sigma, which is the ratio of the scaled network locE and network gE. The scaled network locE (network gE) is the ratio of the network locE (network gE) of the real brain network and mean value of 100 degree-matched random networks (see the formulas in the supplementary materials). Typically, a small-world network has a sigma value greater than 1. For network gE, it measures the capability for parallel information transfer at the level of the entire network and reflects the functional integration of the network^26^, and is defined as the average of the inverse of all shortest path lengths (minimal number of edges that one node must traverse to reach another) in a given network. The network locE is defined as the average of the global efficiency of each node’s neighborhood sub-graph. Note that the network locE is not the most direct measure of network segregation, but reflects the relative functional segregation in the context of determining the small-worldness together with global-efficiency.

The data preprocessing was performed using SPM (http://www.fil.ion.ucl.ac.uk/spm) and Data Processing Assistant for Resting-State fMRI software (DPARSF^63^; available at http://rfmri.org/DPARSF). The network construction and analyses were performed using the Graph-theoretical Network Analysis Toolkit (GRETNA^64^; available at http://www.nitrc.org/projects/gretna/).

### SVR Lesion models

#### Lesion pattern and features

We calculated nodal lesion percentage (*Lp*) in each of the 180 cortical node to quantify a patient’s lesion pattern. For each node, the lesion percentage (*Lp*) was calculated:

1 $$Lp=\frac{{N}_{lesionvoxlesoverlapedwiththenode}}{{N}_{voxelsizeofthenode}}$$

#### SVRlesion model

*SVR lesion model*. We performed a linear SVR analysis with the default parameters (LIBSVM, http://www.csie.ntu.edu.tw/~cjlin/libsvm/) on each of the three network attributes. For a linear model, SVR can be described as

2 $${\rm{Y}}={\omega }^{T}\phi (X)+b$$

where *X* is the lesion patterns across patients, *Y* is network attribute, *φ*(*X*) is the function transforming the lesion patterns to a higher dimensional feature space, ω = (*ω*_0_, *ω*_1_, *ω*_2_, …)^T^ is the fitting coefficient (weight) in the high dimensional space, and *b* is the fitting error.

Two nodes were not included because no patient had any lesion on them: the left occipital fusiform gyrus and the right temporal fusiform cortex.

#### The effect of confounding factors

Some potential confounding variables, age, time after lesion and total lesion volume, have been showed to be predictive for network topological metrics^52,65^. In this study, small but significant Pearson correlation coefficients were observed between total lesion volume and network locE (*r* = 0.236, *P* = 0.021) and gE (*r* = 0.226, *P* = 0.027), and between age and network locE (*r* = −0.201, *P* = 0.049) and sigma (*r* = −0.263, *P* = 0.001) (Table S2 and Fig. S12). No significant correlation was found between the network metrics and time after lesion (*Ps* > 0.17; Table S2). However, the prediction weights of these two factors were not significant in the SVR models.

#### Prediction accuracy and significance

In each turn of the LOOCV, one patient was designated as the test sample and the remaining patients were used to train the lesion model. The predicted score was then obtained by the feature matrix of the tested sample. After all LOOCV rounds were completed, the Pearson correlation coefficients between the predicted and actual network attributes were computed to generate the predictive accuracy (Fig. 1).

Statistical significance of the predictive accuracy was determined using 1000 nonparametric permutation tests. For each permutation test, the prediction labels (i.e., the patients’ network attributes) were randomized, and the same SVR prediction process as used in the actual data was carried out. After 1000 permutations, a random distribution of accuracies was obtained and the *P* value was correspondingly calculated:

3 $$P=\frac{({\rm{number}}\,{\rm{of}}\,{\rm{permutation}}\,{\rm{accuracies}}\, < \,{\rm{actual}}\,{\rm{accuracy}})+1}{{\rm{number}}\,{\rm{of}}\,{\rm{permutations}}+1}$$

### Lesion hub detection based on the predictive weight

We defined those features with significant weights, determined by permutation testing, as lesion hubs. The random distributions of feature weights were obtained according to the previous 1000 permutations. The significance of the feature weight was set at the < 2.5 or > 97.5 percentiles (i.e., *P* < 0.05 for a two-tailed test). For significant lesion models, regions with significant weights were mapped onto the cortical surfaces using the BrainNet Viewer package^66^ (available at http://www.nitrc.org/projects/bnv/).

For methods of healthy connectome topological property calculation and validation analyses, see Supplementary Materials.

### Data Availability

The datasets generated during and/or analysed during the current study are available from the corresponding author on reasonable request.

## Electronic supplementary material

Supplementary Materials
