# Supplementary material for: Brain hubs in lesion models: Predicting functional network topology with lesion patterns in patients
Source: Sci Rep. 2017 Dec 20;7:17908. doi: 10.1038/s41598-017-17886-x (PMC5738424; doi:10.1038/s41598-017-17886-x)
Supplement: Supplementary file 1 — Supplementary Materials [file 41598_2017_17886_MOESM1_ESM.pdf]

Brain hubs in lesion models:

Predicting functional network topology with lesion patterns in patients

Binke Yuan, Yuxing Fang, Zaizhu Han, Luping Song, Yong He & Yanchao Bi

Supplementary Figures

Figure S1. Overall lesion distribution of the 96 patients: number of patients having lesion in each voxel (a) or of the 180 regions (b).

Figure S2. The scatter plots of actual network topological properties versus predicted values using the three lesion models and the corresponding linear regression line. The *r* values were the Pearson correlation coefficients between the predicted values and the actual values, and the *P* values (one-tailed) were calculated based on 1000 permutation tests.

Figure S3. Module organization, nodal WMD and PC of the averaged whole-brain healthy functional network (*N* = 144). (a and b) The module assignments and modularity Q values in sparsity range 0.05–0.2 (step 0.01). Four modules, the visual network, frontal-parietal network, default mode network and somatosensory network, were consistently identified. A representative sparsity threshold, *s* = 0.15 was selected to calculated the thresholded binary functional network (c), the module organization (d, *Q* = 0.47, *Z-score* = 63.76), PC (e) and WMD (f). (g) Scatter plot showing node roles based on nodal PC and WMD .

Figure S4. Nodal gE of the averaged whole-brain healthy functional network (*N* = 144).

Figure S5. Results of lesion hubs when network metrics were calculated using AUC in sparsity thresholds 0.13–0.47 (step 0.01).

Figure S6. Results of lesion hubs when network metrics were calculated based on censored data. One subject who had fewer than 50 volumes after scrubbing was removed.

Figure S7. Results of lesion hubs when lesion pattern and network metrics were calculated using the Brainnetome Atlas.

Figure S8. Results of lesion hubs when lesion pattern and network metrics were calculated using the Craddock 1000 Atlas. Note that because the high non-independence among features when using the Craddock atlas with 1000 partitions, we focused on the lesion-hub detection results with the Craddock 200 atlas and the Brainnetome atlas.

Figure S9. The lesion overlap of 11 patients with focal brain lesion in the left insula (a lesion hub with "integration" lesion effect identified in lesion models).

Figure S10. The changing directions of network topology after focal brain lesion in the left insula. Two-sample t-tests between the 11 patients and 36 matched controls were performed and confounding variables (age, sex and education) were considered. The network locE and gE were normalized by 100 degree-matched random networks. The imbedded scatter plots show the distributions (mean and standard deviations of each group and the *t* and *p* values of Two-sample t-test) of network topological properties in $s = 0.15$.

Figure S11. The averaged BOLD tSNR (temporal signal noise ratio) of the 36 controls. The tSNR for each node was estimated using the normalized data: $tSNR = {S_{mean}}/{\delta_{mean}}$, where the S_{mean} and δ_{mean} are the mean intensity of the averaged BOLD signals and the standard deviation of the averaged BOLD signals of

a node, respectively. (a) The mean tSNR averaged across the 36 controls. (b) The histogram of the averaged tSNR. (c) Most nodes satisfy the criteria for signal quality (mean tSNR > 80).

Figure S12. The relationship between patient's age and the network topological properties (*s* = 0.15).

Supplementary Tables

Table S1. Detailed demographic and etiological characteristics of the 96 patients.

Table S2. Pearson's correlation coefficient between topological metrics (*s* = 0.15) and potential confounding variables for all subjects.

Table S3. SVR prediction accuracies for the validation analyses.

Table S4. Participants' demographics of the 11 patients with focal brain lesions in the left insula and 36 matched controls.

Supplementary Methods

- Network attributes calculation

- Calculation of nodal centrality measures in the healthy functional network

-Validation analyses

Supplementary Figures

Figure S1

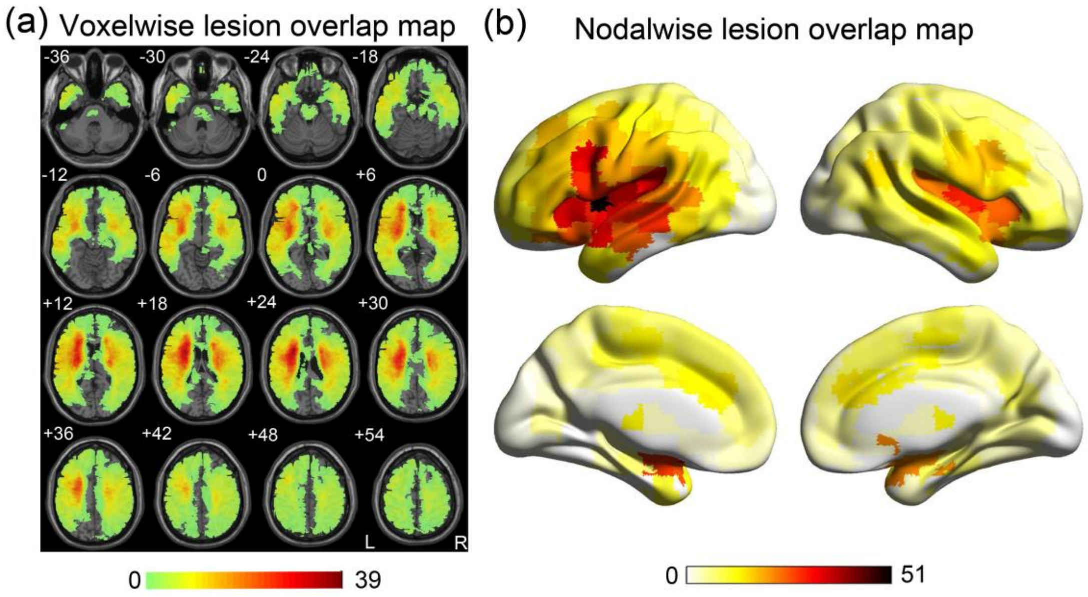

Figure S1. Overall lesion distribution of the 96 patients: number of patients having lesion in each voxel (a) or of the 180 regions (b).

Figure S2

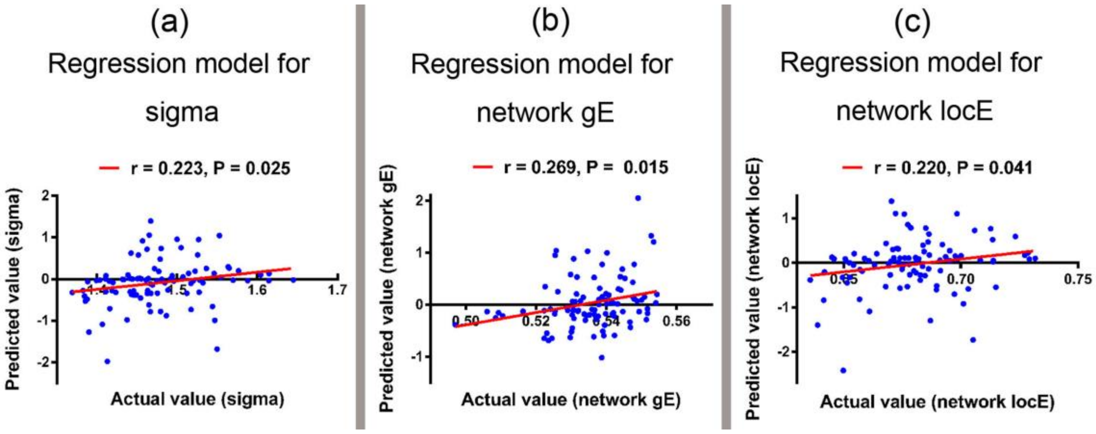

Figure S2. The scatter plots of actual network topological properties versus predicted values using the three lesion models and the corresponding linear regression line. The *r* values were the Pearson correlation coefficients between the predicted values and the actual values, and the *P* values (one-tailed) were calculated based on 1000 permutation tests.

Figure S3

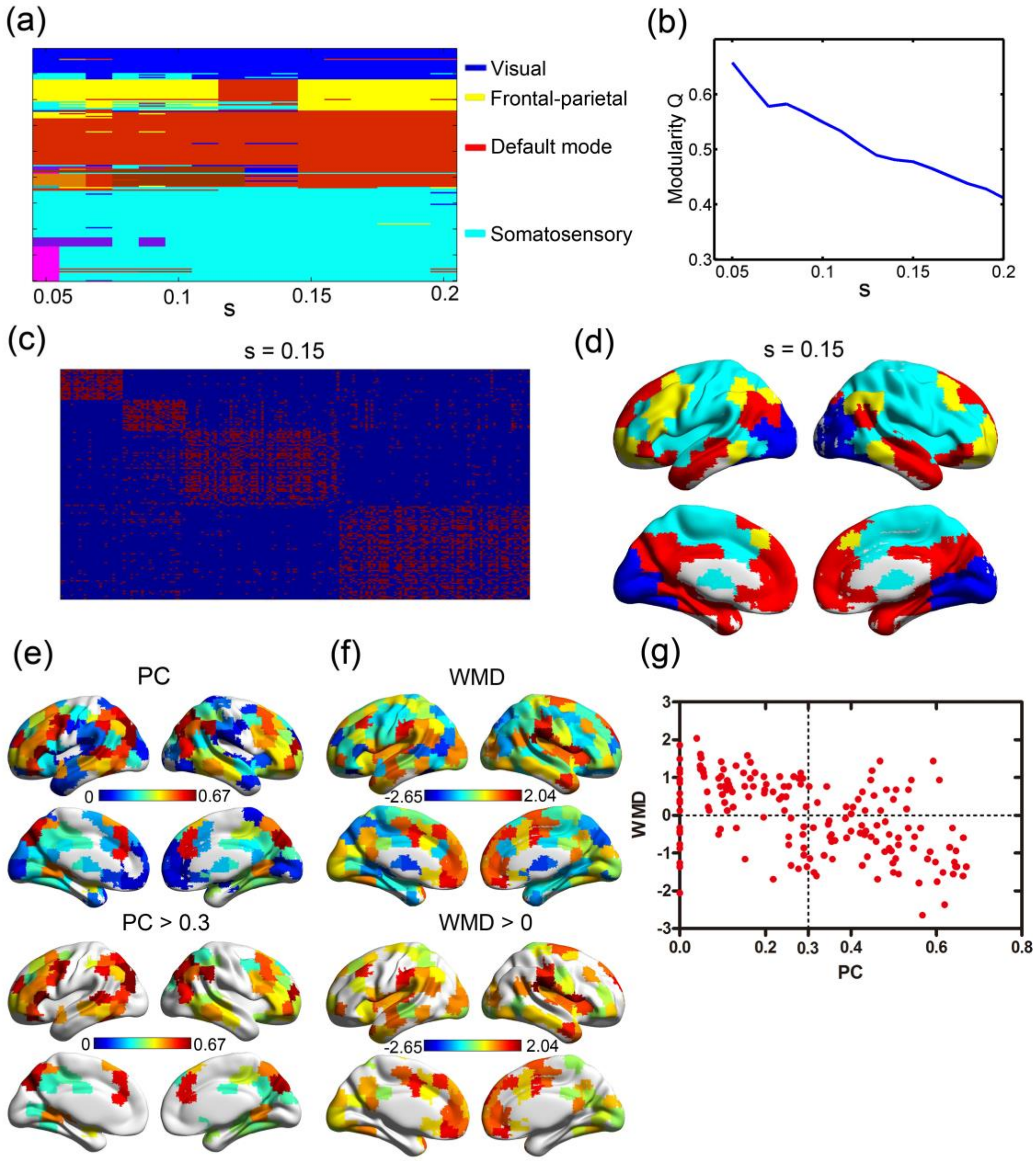

Figure S3. Module organization, nodal WMD and PC of the averaged whole-brain healthy functional network (*N* = 144). (a and b) The module assignments and modularity Q values in sparsity range 0.05–0.2 (step 0.01). Four modules, the visual network, frontal-parietal network, default mode network and somatosensory network, were consistently identified. A representative sparsity threshold, *s* = 0.15 was selected to calculated the thresholded binary functional

network (c), the module organization (d, *Q* = 0.47, *Z-score* = 63.76), and PC (e) and WMD (f). (g) Scatter plot showing node roles based on nodal PC and WMD .

Figure S4

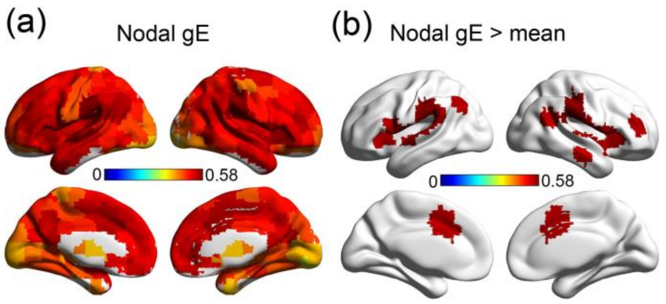

Figure S4. Nodal gE of the averaged whole-brain healthy functional network (*N* = 144).

Figure S5

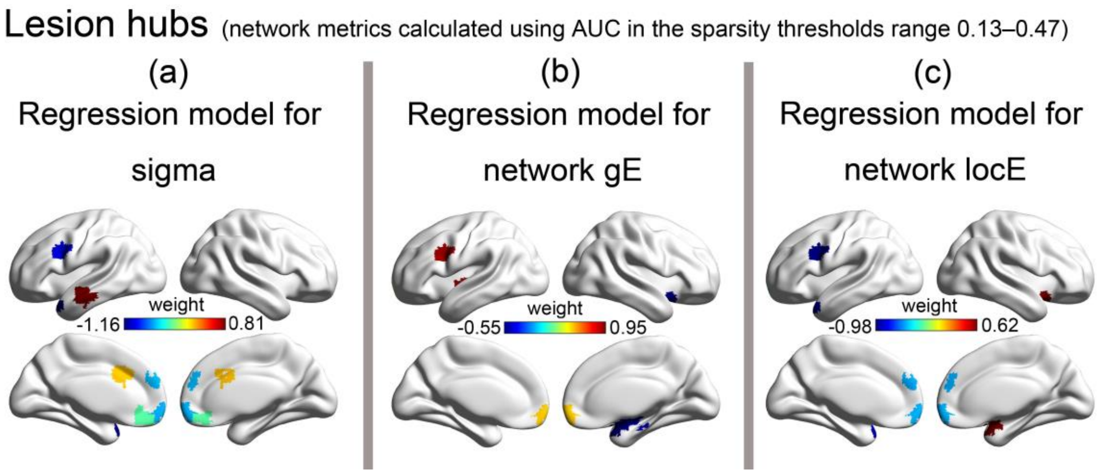

Figure S5. Results of lesion hubs when network metrics were calculated using AUC in sparsity thresholds 0.13–0.47 (step 0.01).

Figure S6

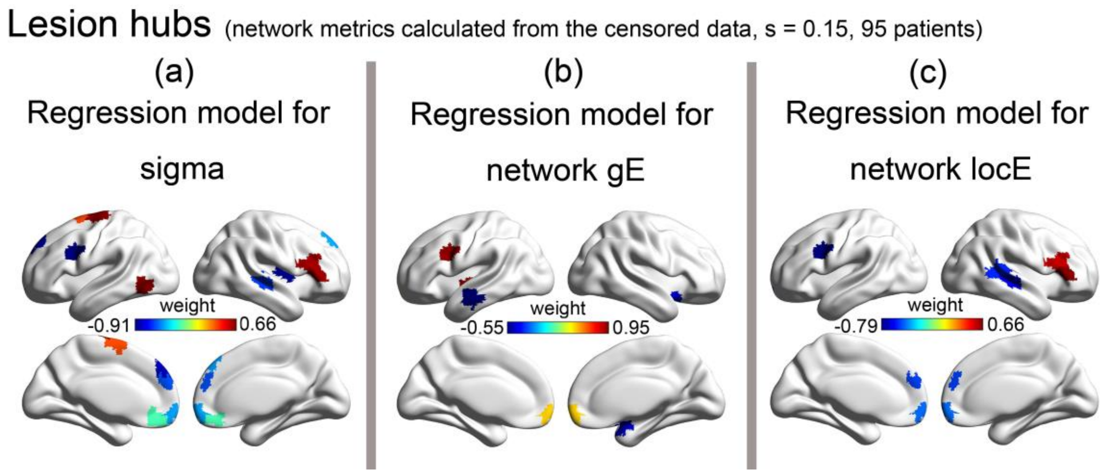

Figure S6. Results of lesion hubs when network metrics were calculated based on censored data. One subject who had fewer than 50 volumes after scrubbing was removed.

Figure S7

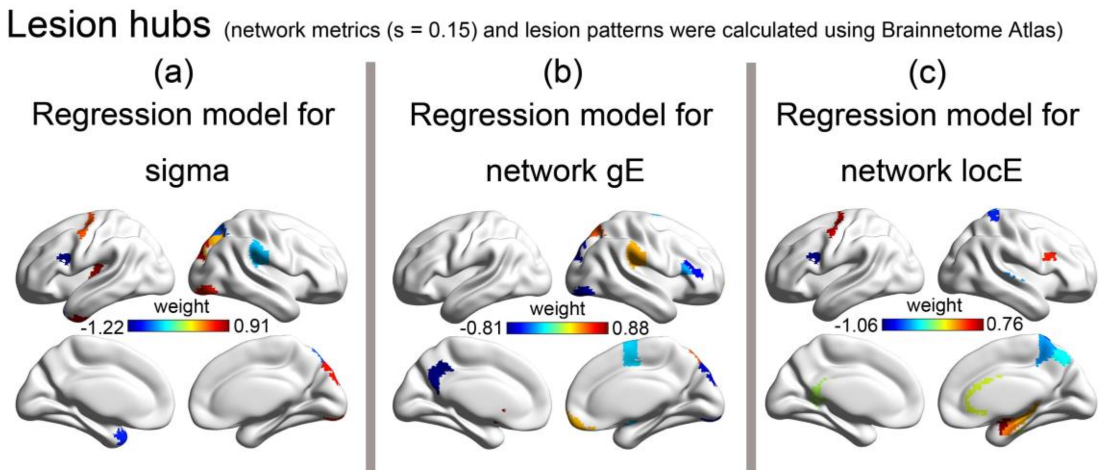

Figure S7. Results of lesion hubs when lesion pattern and network metrics were calculated using the Brainnetome Atlas.

Figure S8

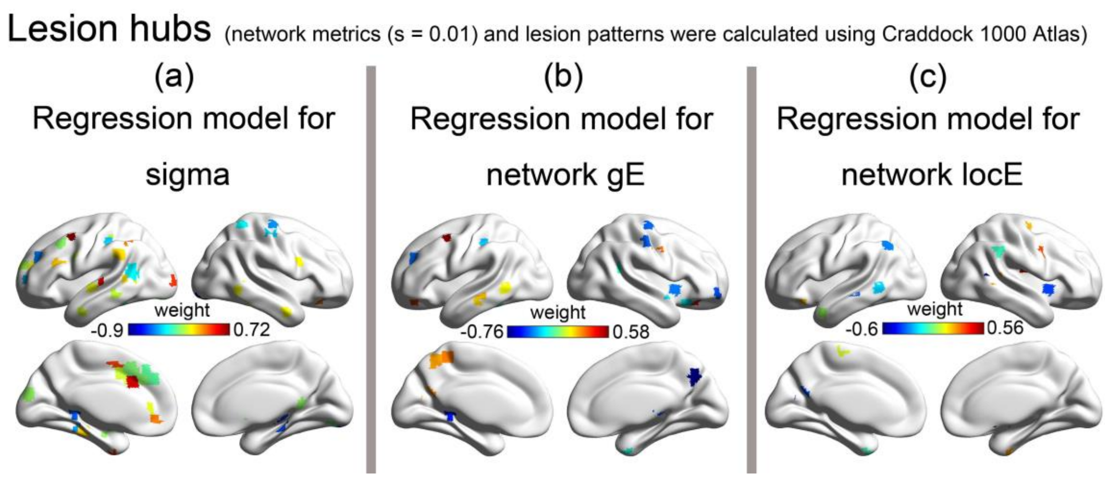

Figure S8. Results of lesion hubs when lesion pattern and network metrics were calculated using the Craddock 1000 Atlas. Note that because the high non-independence among features when using the Craddock atlas with 1000 partitions, we focused on the lesion-hub detection results with the Craddock 200 atlas and the Brainnetome atlas.

Figure S9

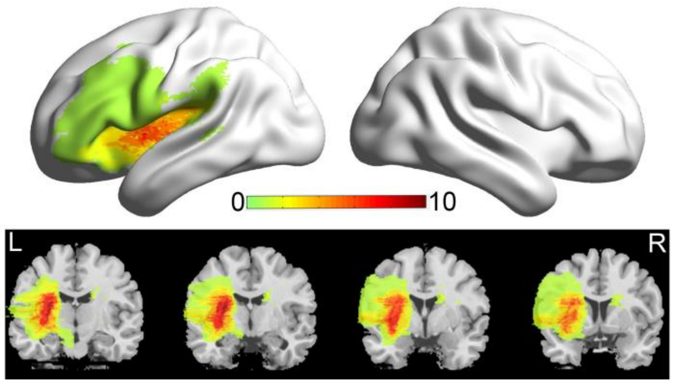

Figure S9. The lesion overlap of 11 patients with focal brain lesion in the left insula (a lesion hub with “integration” lesion effect identified in lesion models).

Figure S10

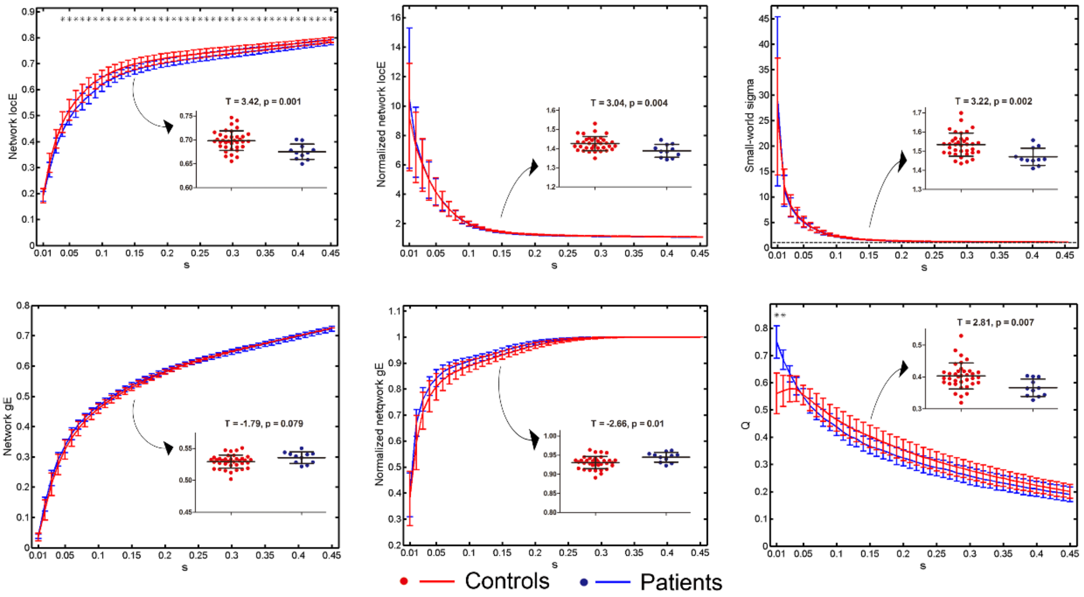

Figure S10. The changing directions of network topology after focal brain lesion in the left insula. Two-sample t-tests were performed between the topological properties of 11 patients and 36 controls that were matched on demographic variables (age, sex and education). The network locE and gE were normalized by 100 degree-matched random networks. The imbedded scatter plots show the distributions (mean and standard deviations of each group and the *t* and *p* values of Two-sample t-test) of network topological properties in *s* = 0.15.

Figure S11

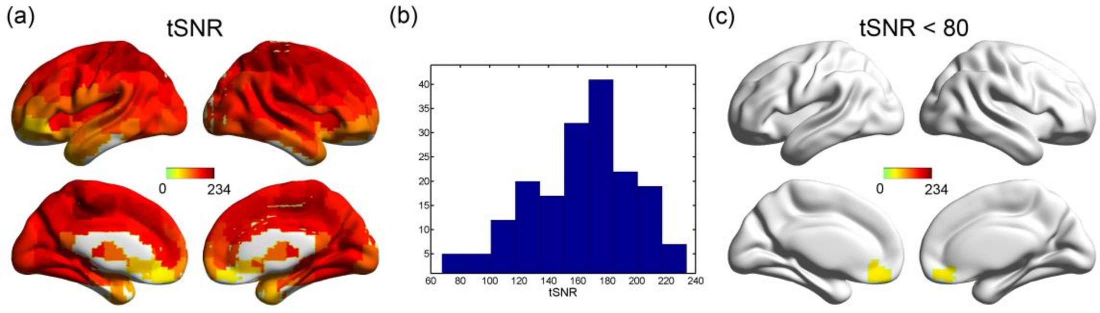

Figure S11. The averaged BOLD tSNR (temporal signal noise ratio) of the 36 controls. The tSNR for each node was estimated using the normalized data: $tSNR = {}^{S_{mean}}/_{\delta_{mean}}$, where the S_{mean} and δ_{mean} are the mean intensity of the averaged BOLD signals and the standard deviation of the averaged BOLD signals of a node, respectively. (a) The mean tSNR averaged across the 36 controls. (b) The histogram of the averaged tSNR. (c) Most nodes satisfy the criteria for signal quality (mean tSNR > 80).

Figure S12

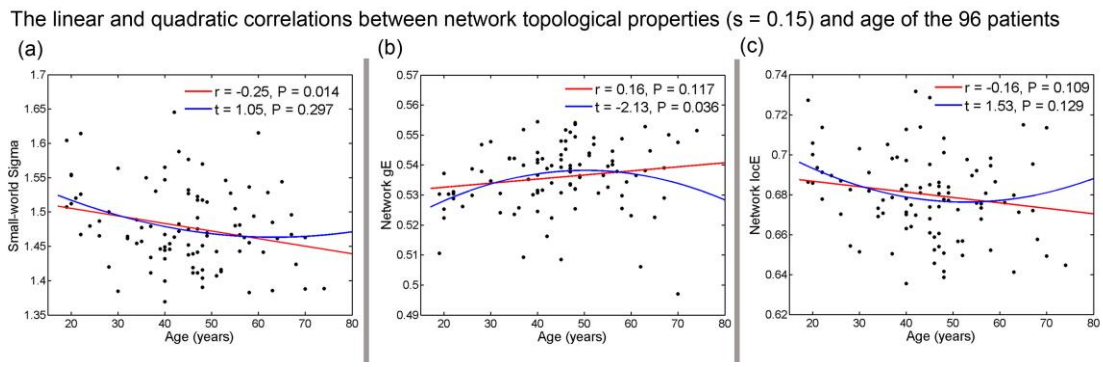

Figure S12. The relationship between patient's age and the network topological properties (*s* = 0.15).

Supplementary Tables

Table S1. Detailed demographic and etiological characteristics of the 96 patients

Code	Sex	Age (year)	Education (year)	Time since injury (months)	Cause of disease	Lesion site				
						Left hemisphere		Right hemisphere		Brain stem
						GM	WM	GM	WM	
4	male	49	16	6	hemorrhage	F, P, T, Ins, BG	*	-	-	-
7	male	48	15	4	infarction	Lim, BG	*	-	*	-
8	male	60	16	6	hemorrhage	F, P, T, Lim, Ins, BG	*	F, Lim	*	-
9	male	43	19	11	infarction	-	-	Lim, BG	*	-
15	male	22	12	87	trauma	P, O, Lim	*	-	-	-
16	male	32	15	9	hemorrhage	F, O, T, Lim, Ins, BG	*	-	-	-
17	female	26	9	10	atrophy	F, P, O, Lim	*	-	-	-
18	male	34	12	10	hemorrhage	F, P, T, Lim, Ins, BG	*	-	-	-
19	male	42	14	6	infarction	F, P, T, Lim, Ins, BG	*	-	-	-
21	male	63	12	1	infarction	-	-	P, Ins, Lim, Thal	*	-
22	male	49	12	3	infarction	F, P, T, Ins, Lim, BG, Thal	*	-	-	-
25	male	46	16	2	infarction	-	-	O, T, BG, Thal	*	-
26	male	59	4	10	gas poisoning	Lim, BG	-	F, Lim, BG	*	-
27	male	58	12	6	unknown	-	-	-	-	*
28	male	32	12	9	electric shock	F, P, Lim, BG	*	-	*	-
29	male	30	19	3	infarction	-	-	F, P, T, Lim, Ins, BG, Thal	*	*
30	male	41	15	8	hemorrhage	T, Ins, Lim, BG, Thal	*	-	-	-
33	male	36	15	10	hemorrhage	F, Ins, BG, Thal	*	-	-	-
34	female	64	12	5	infarction	F, Ins, BG, Thal	*	-	-	-
35	male	46	12	6	infarction	BG	*	BG, Thal	*	-
38	male	40	12	3	hemorrhage	-	-	F, T, Ins, Lim, Thal	*	-
42	male	45	12	3	hemorrhage	F, Ins, BG	*	F, P, T, Lim, BG	*	*
48	female	56	12	6	hemorrhage	-	-	F, Ins. BG, Thal	*	*
56	male	61	15	15	hemorrhage	F	*	F, O, Ins, BG	*	-
57	male	51	9	2	infarction	F, P, T, Ins, Lim	*	-	-	-
60	male	45	16	4	infarction	T, Lim, Ins, BG, Thal	*	-	-	*

62	female	56	12	6	infarction	F, P, O, T, Ins, BG	*	-	-	-
66	male	35	16	2	infarction	-	-	F, Lim, BG, Ins, Thal	*	-
67	male	47	9	5	infarction	-	-	F, P, Ins, Lim, BG, Thal	*	*
68	male	40	16	6	hemorrhage	F, P, T, Lim, Ins, BG, Thal	*	BG	*	*
78	male	40	8	2	trauma	T	*	F, T	*	-
79	male	48	19	24	hemorrhage	-	*	P, F, O, T, Lim, Ins, BG, Thal	*	*
83	male	54	16	4	infarction	-	-	F, P, O, Lim, Thal	*	-
85	male	67	9	7	infarction	F, P, T, Ins, Lim, BG	*	-	-	-
86	male	67	12	5	thrombosis	F, Lim, Ins, BG	*	F, Lim, BG	*	-
87	female	45	9	30	trauma	BG, Thal	*	F, P, T, Lim, Ins, BG	*	*
88	female	70	16	3	trauma	F, P, T, Lim, Ins	*	F, BG, L,Ins	*	-
89	male	46	9	2	infarction	F, P, T, Lim, BG	*	F, P, Lim, BG	*	-
91	male	65	9	8	infarction	F, P, T, Lim, BG, Thal	*	F, P, O, T, Lim, Ins, BG	*	*
97	male	55	15	3	infarction	F, Ins, BG	*	F, P, T, Lim, BG, Thal	*	*
98	male	62	12	10	infarction	Ins, Lim, BG	*	F, P, Lim, BG	*	-
101	male	35	16	2	infarction	F, P, T, Lim, BG	*	-	-	-
102	male	28	16	3	infarction	F, P, O, T, Ins, BG	*	-	-	-
103	female	37	12	3	infarction	F, P, O, T	*	-	-	-
104	male	68	16	2	infarction	F, P, O, T, BG, Thal	*	BG	*	-
106	female	22	16	2	hemorrhage	-	*	F, P, Lim, BG	*	-
109	male	58	9	7	infarction	F, P, Lim, Ins, BG	*	BG	*	-
111	female	51	8	5	infarction	Ins, BG	*	F, P, O, T, Lim, Ins, BG	*	-
112	male	22	15	11	trauma	T, Ins, BG	*	-	-	-
114	male	22	15	2	trauma	F, P, O, T, Lim	*	-	-	-
115	male	20	9	2	hemorrhage	F, O, T	*	F, T	*	-
116	male	56	15	5	infarction	F, P, T, Lim, Thal	*	-	-	*
117	female	45	15	2	infarction	-	-	F, P, O, T, Lim, Ins, BG	*	-
118	female	40	12	6	infarction	P, F, T, Ins, BG, Thal	*	-	-	-
122	male	63	12	2	infarction	F, P, O, T, Lim, Ins, BG, Thal	*	-	-	-
123	male	24	9	2	trauma	-	-	P, F, Lim, Ins	*	-
124	male	52	12	3	infarction	F, P, T, Lim, Ins,	*	-	*	-

						BG, Thal				
125	male	48	9	3	hemorrhage	-	*	P, F, O, T, Lim, Ins, BG	*	*
126	male	48	9	2	infarction	-	-	F, P, T, O, Lim, Ins, BG, Thal	*	*
129	male	58	15	1	infarction	F, P, Lim, Ins, BG	*	-	-	-
132	male	37	12	3	infarction	F, P, O, T, Ins, Lim, BG, Thal	*	-	*	-
133	male	47	15	3	infarction	F, P, T, Lim, Ins, BG, Thal	*	-	-	*
137	female	40	12	3	trauma	BG	*	F	-	-
141	male	19	12	3	trauma	T, Thal	*	P	*	-
159	female	39	15	3	hemorrhage	-	-	T, Lim, Ins, BG, Thal	*	-
161	male	53	12	1	infarction	Lim, P, Thal	*	F, P, O, T, Lim, Ins, BG	*	-
162	male	48	15	3	hemorrhage	F, P, O, T, Ins, BG	*	-	*	-
163	male	47	16	2	infarction	F, BG, Ins	*	BG	*	-
165	male	47	16	5	hemorrhage	F, P, T, Ins, BG	*	F, BG	*	-
170	male	43	16	16	infarction	F, T, Lim, Ins, BG	*	F, P, Lim, BG	*	-
175	male	20	15	1	trauma	P, T	*	-	-	-
177	male	26	16	3	infarction	F, P, T, Lim, Ins, Thal	*	-	-	-
180	female	41	15	5	hemorrhage	F, P, T, Ins, BG	*	-	-	-
181	male	49	15	5	hemorrhage	BG, Ins	*	F, P, T, Lim, BG, Thal	*	*
182	female	40	15	3	infarction	F, P, O, T, BG, Thal	*	F, P, Lim, T, Ins, BG	*	-
185	male	57	6	2	infarction	Ins, BG, Thal	-	-	*	-
190	male	42	12	6	hemorrhage	Lim, BG, Thal	*	-	-	-
191	male	28	16	3	hemorrhage	F, P, T, Thal, BG	*		-	-
192	male	21	12	48	trauma	Ins, BG	*	F, BG	*	*
194	male	40	16	2	hemorrhage	F, Ins, BG	-	F, P, T, Ins, Thal, BG	*	*
195	male	30	12	2	trauma	F, Lim	-	F, Lim	-	-
204	male	38	12	3	infarction		-	Lim, BG, Thal		-
205	male	47	12	2	trauma	-	-	BG, Ins, Thal	*	-
206	male	40	15	2	hemorrhage	T, Ins, BG, Thal	*	-	-	*
208	male	45	9	2	trauma	-	-	P, T	-	-
209	male	48	9	2	hemorrhage	P, F, T, Lim, Ins, BG	*	F, Ins, BG	*	*
210	male	74	12	3	hemorrhage	F, BG	*	F, P, O, T, Lim, BG	*	-
211	female	52	6	3	infarction	-	-	P, F, T, Lim, BG	*	-
213	male	55	15	2	hemorrhage	P, T, O, Ins	*	-	-	-

214	female	19	12	2	trauma	P, O, T	*	-	-	-
217	female	34	15	2	infarction	P, F, O, T, Ins, BG	*	-	-	-
222	female	37	9	7	trauma	BG	-	-	-	-
226	male	45	15	5	infarction	F, P, T, Lim, Ins, BG	*		-	-
231	male	43	15	4	infarction	F, Ins	-	-	-	*
304	male	70	12	2	infarction	F, P, O, T, Lim, Ins, BG	*	F, BG	*	-
305	male	47	12	4	infarction	F, Ins, BG	*	-	-	-

Note: LH: left hemisphere; RH: right hemisphere; F: frontal lobe; P: parietal lobe; O: occipital lobe; T: temporal lobe; Lim: limb lobe; Ins: insula; BG: basal ganglia; Thal: thalamus; *: had lesion; -: no lesion.

Table S2. Pearson's correlation coefficient between topological metrics (*s* = 0.15) and relevant variables for all subjects

	Correlation coefficient/ *P* value	Network LocE	Network gE	Sigma	Age	Time after lesion	Total lesion volume
Network LocE	*r*		**-0.765**	**0.915**	**-0.201**	0.065	**-0.329**
	p		**1.24E-19***	**6.22E-39***	**0.049**	0.529	**0.001**
Network gE	*r*			**-0.553**	0.118	-0.018	0.015
	p			**5.34E-9***	0.253	0.862	0.888
Sigma	*r*				**-0.263**	0.091	**-0.31**
	p				**0.001**	0.38	**0.002**
Age	*r*					-0.187	0.113
	p					0.069	0.273
Time after lesion	*r*						-0.112
	p						0.276
Total lesion volume	*r*						
	p						

* $P < 0.05$

Table S3. SVR prediction accuracies for the validation analyses

Topology	Correlation coefficient/ P value	AUC	Scrubbing	76 stroke patients	77 male patients	No GSR	Brainnetome atlas	Craddock 1000 atlas	Split-half reproducibility assessment	
									Sub group1	Sub group2
Sigma	*r*	**0.29**	**0.241**	0.049	0.106	0.093	**0.449**	**0.188**	0.185	**0.425**
	p	**0.013***	**0.033***	0.194	0.117	0.136	**< 0.001***	**0.045***	0.091	**0.013***
Network gE	*r*	**0.287**	**0.246**	**0.222**	**0.236**	0.168	**0.454**	**0.267**	**0.383**	**0.402**
	p	**0.012***	**0.024***	**0.034***	**0.03***	0.067	**< 0.001***	**0.016***	**0.006***	**0.007***
Network locE	*r*	**0.317**	**0.244**	0.199	**0.278**	**0.249**	**0.387**	**0.319**	**0.348**	**0.392**
	p	**0.007***	**0.019***	0.051	**0.02**	**0.019***	**0.002***	**0.005***	**0.018***	**0.013***

* $P < 0.05$, uncorrected, one-tailed; AUC: Area under the curve; GSR: global signal regression.

Table S4. **Participants' demographics of the 11 patients with focal brain lesions in the left insula and 36 matched controls.**

	Patients	Healthy controls	p-value
Sample size	11	36	
Gender (male/female)	10/1	25/11	0.16[1]
Age (years)	44.64 ± 12.19 (21–64)	48.28 ± 11.98 (26–72)	0.38[2]
Education (years)	13.73 ± 3.07 (6–16)	13.14 ± 4.16 (6–22)	0.67[2]

[1] Pearson Chi-square test; [2] Two-sample t-test.

Supplementary Methods

Network attributes calculation

(1&2) Local and global network efficiency

The local and global network efficiencies of a network measure the network's capability for information transmission at the local and global levels, respectively. The "global efficiency" is defined as the inverse of all shortest path lengths in a given network. The global efficiency is defined as follows:

$$E_{global} = \frac{1}{n} \sum_{i \in N} \frac{\sum_{j \in N, j \neq i} d_{ij}^{-1}}{n-1} \quad (1)$$

where $d_{ij} = \sum_{a_{uv} \in g_{i \leftrightarrow j}} a_{uv}$, $g_{i \leftrightarrow j}$ is the shortest path between *i* and *j*. a_{uv} is the connection status between *u* and *v*, i.e., 1 for presence of connection and 0 for absence.

The "local efficiency" of a node is defined as follows:

$$E_{loc} = \frac{1}{N} \sum_{i \in N} \frac{\sum_{j,h \in N, j \neq i,} a_{ij} a_{ih} [d_{jh}(N_i)]^{-1}}{k_i\,(k_i - 1)} \quad (2)$$

where $d_{jh}(N_i)$ is length of the shortest path between *j* and *h*, that contains only neighbors of *i*, k_i is the degree of node *i*, i.e., number of links connected to *i*.

(3) Small-world

Small-world is a measure of a network that incorporates the two abovementioned efficiency metrics. In this study, small-worldness was calculated based on the ratios of the scaled local efficiency ($E_{loc}/E_{loc-rand}$) and scaled global efficiency ($E_{glob}/E_{glob-rand}$) between the real brain functional networks and 100 degree-matched random networks:

$$Sigma = \frac{E_{loc}/E_{loc-rand}}{E_{glob}/E_{glob-rand}} \quad (3)$$

Typically, if a network has a higher local efficiency than its random counterparts ($E_{loc}/E_{loc-rand} > 1$) and an approximately equivalent global efficiency ($E_{glob}/E_{glob-rand} \approx 1$), i.e., sigma > 1, the network is said to demonstrate "small-worldness".

Calculation of nodal centrality measures in the healthy functional network

We calculated the nodal attributes on a healthy dataset including 144 healthy college students reported in our previous study[1]. All 144 subjects were recruited from Beijing Normal University. They were all right-handed, with no history of neurological or psychiatric disorders. All participants provided written informed consent. Scanning was performed on a 3.0 T Siemens Tim Trio scanner at the Beijing Normal University Imaging Centre for Brain Research. High-resolution three-dimensional T1-weighted magnetization-prepared rapid gradient-echo images were acquired for anatomic reference (repetition time (TR) = 2530 ms, echo time (TE) = 3.39 ms, inversion time (TI) = 1100 ms, flip angle (FA) = 7°, 144 sagittal no-gap slices, voxel size = 1.33 × 1 × 1 mm^3, field of view (FOV) = 256 × 256 mm). Resting-state images were obtained using a gradient-echo echo-planar sequence sensitive to blood oxygenation level-dependent contrast (TR = 2000 ms, TE = 30 ms; FA = 90°, 33 axial slices acquired interleaved with a 0.7 mm gap, voxel size = 3.125 × 3.125 × 4.2 mm^3, FOV = 200 × 200 mm, 200 volumes). Participants were instructed to stay awake and keep their eyes closed during the functional runs.

The preprocessing procedures included slice timing, realignment, registration, detrending, filtering (0.01–0.1 Hz), and nuisance covariance regression (Friston 24 head motion, global signal, cerebrospinal fluid, and white matter). The network construction was the same as that of patient data. The functional connectivity matrix for each youth participant was first *z*-transformed using Fisher z-transformation and then averaged across subjects. The mean functional connectivity matrix was used for graph theory analysis. Three nodal attributes were calculated: global efficiency (gE), participant coefficient (PC) and within-module degree (WMD).

The calculation formulas of nodal gE were the same as those described above.

Next, we provided detailed descriptions of nodal PC and WMD. The two nodal attributes relied on the module organization of the functional network. Thus, we first defined the modules by performing modularity analysis. Here we used Newman's

modularity algorithm based on a spectral optimization algorithm. For a given network, the modularity Q is:

$$Q = \frac{1}{l}\sum_{i,j\in N}\left(a_{ij} - \frac{k_i k_j}{l}\right)\delta_{m_i m_j}$$

(4)

where m_i is the module containing node i, and $\delta_{m_i m_j}$ = 1 if m_i = m_j, and 0 otherwise.

To validate the robustness of the partition, we calculated the modular assignment of each node in sparsity range 0.05–0.2, with 0.01 increment.

Four modules were consistently defined (Fig. S3 a and b): visual network, default mode network, frontal-parietal network and somatosensory network. Here, we chose a representative sparsity threshold (s = 0.15) to get the modular assignment. Based on this module organization, the PC of a node *i* is defined as follows:

$$PC(i) = 1 - \sum_{m\in M}\left(\frac{k_i\,(m)}{k_i}\right)^2 \quad (5)$$

where M is the number of modules, $k_i\,(m)$ is the number of links between node *i* and all nodes in module *m* and k_i is the total degree of node *i*. The $PC(i)$ tends towards 1 if node *i* has a homogeneous connection distribution with all the modules and towards 0 if it does not have any inter-module connections. PC measures the ability of a node to maintain communication between its own module and the other modules. A high PC value for a given node usually means the node has many inter-module connections.

The WMD score is a z-scored measure of the number of intramodule connections to each node. The WMD of node *i* is defined as follows:

$$WMD(i) = \frac{k_i - \bar{k}_{is}}{\delta_{is}} \quad (6)$$

where $\bar{k}_{is}$ is the average degree of all nodes in module *s* and δ_{is} is the standard deviation of the degree of all nodes in module *s*.

<u>Validation analyses</u>

A series of validation analyses were performed to examine the robustness of the

main results. Details are described below for each of these validations:

1) *The effect of network sparsity threshold*. We used the AUC to calculate network metrics across the 0.13–0.47 (step 0.01) sparsity range and SVR analyses were then reanalyzed;

2) *Head motion*, we further performed a "scrubbing" procedure on the preprocessed images. For each participant, preprocessed 4D data were first censored based on a criterion of framewise displacement > 0.5 mm (but with at least 50 time points left). The network attribute were calculated using these censored volumes and SVR analyses were then re-conducted;

3) *The effect of global signal removal*. Global signal removal is a controversial preprocessing step[2]. It was performed in the main analysis, but we also reanalyzed our data without regressing out the global signal;

4) *The effect of brain parcellation*. We repeated the whole analyses using another brain parcellation – Brainnetome Atlas[3] (246 nodes) – to investigate the sensitivity of prediction to the choice of parcellation scheme. We chose this atlas because its parcellation was done on the basis of structural connectivity and had comparable number of regions with the Craddock 200 atlas. We also considered a third Craddock 1000 atlas[4] which contained much finer parcellations. For Craddock 1000 atlas, after excluding the nodes in cerebellum and brainstem, 789 cerebrum nodes were used to generate a finer lesion pattern and to construct functional network. We then reperformed the SVR analysis in sparsity threshold, $s = 0.01$, an arbitrary threshold to ensure sparse and fully-connected networks;

5) *The effects of lesion type and handedness*. To maximize lesion coverage, we included multiple types of patients in the main analysis. To ensure that our results were not contaminated by etiology and handedness, we re-performed the SVR prediction and lesion hub detection on only the 76 right-handed (handedness assessed using the Chinese adaptation of Edinburgh inventory[5]) stroke patients (13 females; age: mean ± SD = 47.013 ± 11.218; range, 20–74 years);

6) *The effect of gender.* The gender effect on small-world brain networks have been showed by previous studies[6,7]. The ratio of male/female of the patient group is

unbalanced, 19/77. To test the extent to which our results are affected by sex, we re-performed the SVR prediction and lesion hub detection using only the male patients (age: mean ± SD = 47.01 ± 11.22; range, 20–74 years);

7) *Validating the feature signs using single-nodal lesion patients*. To test whether positive or negative features imply increased or reduced topological values, 11 patients with focal lesion in the left insula and 36 matched controls (Table S4) were adopted to investigate the changing directions of network topological properties after focal lesion. For controls, the imaging data collection and preprocessing procedures were the same as those for patients. Six network topological metrics, the network locE and gE, the scaled network locE and gE, the small-worldness sigma and the modularity Q[8] were calculated in a wide sparsity range (0.01–0.45, step 0.01). For each metric, Two-sample t-tests were performed and corrected by FDR correction ($P < 0.05$) for multiple comparisons.

1 Xu, Y., Lin, Q., Han, Z., He, Y. & Bi, Y. Intrinsic functional network architecture of human semantic processing: Modules and hubs. *Neuroimage* **132**, 542-555 (2016).

2 Murphy, K. & Fox, M. D. Towards a consensus regarding global signal regression for resting state functional connectivity MRI. *Neuroimage*(2016).

3 Fan, L. *et al.* The Human Brainnetome Atlas: A New Brain Atlas Based on Connectional Architecture. *Cereb Cortex* **26**, 3508-3526 (2016).

4 Craddock, R. C., James, G. A., Holtzheimer, P. E., 3rd, Hu, X. P. & Mayberg, H. S. A whole brain fMRI atlas generated via spatially constrained spectral clustering. *Hum Brain Mapp* **33**, 1914-1928 (2012).

5 Oldfield, R. C. The assessment and analysis of handedness: the Edinburgh inventory. *Neuropsychologia* **9**, 97-113 (1971).

6 Yan, C. G. *et al.* Sex- and Brain Size-Related Small-World Structural Cortical Networks in Young Adults: A DTI Tractography Study. *Cerebral Cortex* **21**, 449-458 (2011).

7 Tian, L. X., Wang, J. H., Yan, C. G. & He, Y. Hemisphere- and gender-related differences in small-world brain networks: A resting-state functional MRI study. *Neuroimage* **54**, 191-202 (2011).

8 Newman, M. E. Modularity and community structure in networks. *Proceedings of the National Academy of Sciences of the United States of America* **103**, 8577-8582 (2006).
